# Exogenous ketosis in patients with type 2 diabetes: Safety, tolerability and effect on glycaemic control

**DOI:** 10.1002/edm2.264

**Published:** 2021-05-20

**Authors:** Adrian Soto‐Mota, Nicholas G. Norwitz, Rhys Evans, Kieran Clarke, Thomas M. Barber

**Affiliations:** ^1^ Department of Physiology, Anatomy and Genetics The University of Oxford University of Oxford Oxford UK; ^2^ Warwickshire Institute for the Study of Diabetes, Endocrinology and Metabolism University of Warwick Coventry UK

**Keywords:** diabetes, exogenous ketosis, ketone ester, ketosis

## Abstract

**Introduction:**

Ketogenic diets have shown to improve glycaemic control in patients with type 2 diabetes. This study investigated the safety, tolerability, and effects on glycaemic control in patients with type 2 diabetes of an exogenous ketone monoester (KE) capable of inducing fasting‐like elevations in serum β‐hydroxybutyrate (βHB) without the need for caloric or carbohydrate restriction.

**Methods:**

Twenty one participants (14 men and 7 women, aged 45 ± 11 years) with insulin‐independent type 2 diabetes, and unchanged hypoglycaemic medication for the previous 6 months, were recruited for this non‐randomised interventional study. Participants wore intermittent scanning glucose monitors (IS‐GM) for a total of 6 weeks and were given 25 ml of KE 3 times daily for 4 weeks. Serum electrolytes, acid‐base status, and βHB concentrations were measured weekly and cardiovascular risk markers were measured before and after the intervention. The primary endpoints were safety and tolerability, with the secondary endpoint being glycaemic control.

**Results:**

The 21 participants consumed a total of 1,588 drinks (39.7 litres) of KE over the course of the intervention. Adverse reactions were mild and infrequent, including mild nausea, headache, and gastric discomfort following fewer than 0.5% of the drinks. Serum electrolyte concentrations, acid‐base status, and renal function remained normal throughout the study. Compared to baseline, exogenous ketosis induced a significant decrease in all glycaemic control markers, including fructosamine (335 ± 60 μmol/L to 290 ± 49 μmol/L, *p* < .01), HbA1c (61 ± 10 mmol/mol to 55 ± 9 mmol/mol [7.7 ± 0.9% to 7.2 ± 0.9%], *p* < .01), mean daily glucose (7.8 ± 1.4 mM to 7.4 ± 1.3 mM [140 ± 23 mg/dl to 133 ± 25 mg/dl], *p* < .01) and time in range (67 ± 11% to 69 ± 10%, *p* < .01).

**Conclusions:**

Constant ketone monoester consumption over 1 month was safe, well tolerated, and improved glycaemic control in patients with type 2 diabetes.

## Abstract 2

This study involved a month of closely supervised exogenous ketosis using a ketone monoester. Additionally, it involved six weeks of continuous glucose monitorization to compare glucose control before, during and after exogenous ketosis. Exogenous ketosis was safe, well‐tolerated and improved glucose control.

## 1 INTRODUCTION

Exogenous ketosis has therapeutic potential across a diverse range of chronic metabolic diseases, including epilepsy, neurodegenerative diseases, heart failure and diabetes.^1^ Very low carbohydrate or ketogenic diets can reverse type 2 diabetes,^2^ and exogenous ketosis without carbohydrate restriction lowers blood glucose. As examples, d‐β‐hydroxybutyrate (βHB) salt infusion lowers blood glucose in both animals^3^, ^4^, ^5^ and humans.^6^ Additionally, the consumption of ketone ester (KE)^6^ or medium chain triglyceride oils^5^ lowers blood glucose, suggesting that ketosis itself has a hypoglycaemic effect that is independent of carbohydrate restriction.

For reasons that are unknown, the glucose‐lowering effect of ketosis appears to be greater in patients with type 2 diabetes (T2D) than in healthy controls,^7^ but both insulin dependent^8^, ^9^ and independent^10^ mechanisms have been proposed. As some patients find carbohydrate restriction difficult leading to low long‐term adherence^11^ and as exogenous ketosis can independently improve glycaemic control (even after a carbohydrate challenge^12^), there exists a clinical niche for a safe exogenous ketogenic intervention in T2D.

The ketone monoester (KE), (*R*)‐3‐hydroxybutyl‐(*R*)‐3‐hydroxybutyrate, is a drink that, after ingestion, is cleaved by gut esterases into equimolar βHB and (*R*)‐1,3‐butanediol. Both molecules enter the portal circulation, and the latter is converted in the liver into βHB.^13^ Thus, each equivalent of KE yields two equivalents of βHB.

The pharmacology and safety of KE has been thoroughly studied in animals^14^ and in healthy human adults.^15^ However, the safety and tolerability of this supplement has not been investigated in patients with T2D. Given the metabolic and physiological peculiarities of this disease, and the potential benefits of exogenous ketosis on glycaemic control, we aimed to study the safety and tolerability of the KE in patients with T2D.

The KE is available as a sports supplement^16^ and carries benefits over other ketogenic supplements. First, it provides enantiomerically pure d‐βHB, which is important because the body primarily uses the d form of βHB and not the l enantiomer. Second, it is salt‐free and so does not impose an undue sodium load, particularly when used chronically. Third, KE can raise βHB in blood to fasting‐like levels of >3 mM within minutes.^6^ Beyond its practical clinical benefits, the KE stands out as a research tool for determining the relative contributions of ketosis versus carbohydrate reduction in a ketogenic diet, which can reverse T2D. Thus, the KE could help generate insights into disease mechanisms and thereby provide an avenue for developing targeted therapies.

## 2 METHODS

### 2.1 Ethics approval

The research protocol, including amendments and consent forms, was approved by the South Central, Oxford B Research Ethics Committee (18/SC/0064). This study was conducted in accordance with the guidelines set forth by the International Conference on Harmonization Guidelines for Good Clinical Practice, and the Declaration of Helsinki regarding the treatment of human subjects in a research study. All participants provided written informed consent before their enrolment. This project was registered with the code ISRCTN12401551.

### 2.2 Recruitment and participant characteristics

Potential participants were invited via paper advertisements placed on the Warwickshire Institute for the Study of Diabetes, Endocrinology and Metabolism (WISDEM). Interested potential participants contacted the research team via email to schedule a visit to determine eligibility. Recruited were 21 patients with T2D aged 18–70 years (inclusive) who had no past or present use of insulin to control glycaemia. Participants had no documented changes in other hypoglycaemic medications for the 6 months prior to the study. If an enrolled participant were prescribed a change in their glycaemic treatment during the study, their participation was discontinued, and they were excluded from the analysis. Also excluded were patients with serum creatinine >1.5 mg/dl, with elevated (3x the normal upper limit) alanine aminotransferase (ALT) or aspartate amino transferase (AST), with scheduled surgical procedures, or who were pregnant, lactating or planning to become pregnant.

### 2.3 Study design

Each participant was asked to wear an IS‐GM (Abbott FreestyleR; Maidenhead, Berks UK ^®^) for 6 weeks. Week 1 served as a baseline. During weeks 2–5, participants were asked to measure out 25 ml (25 g) of the ketone monoester in a measuring cup and drink it either neat or diluted with the zero calorie artificially sweetened drink of their choice, three times daily. Figure S1 illustrates these interventions. Participants were also asked to record their meal timings, sleep duration, physical activity and symptoms (both positive and negative) in a diary. They were instructed to keep their usual diet and physical activities throughout the study and to allow at least 4 h between KE drinks to elevate serum levels of βHB for at least 12 h every day.

### 2.4 Metabolites and measurements

Tolerability was assessed with self‐recorded open adverse effects diaries following each of the KE drinks. If an adverse effect was registered, participants were asked to rank it as ‘mild’, ‘moderate’ or ‘severe’.

Safety was assessed weekly by measuring peak βHB blood levels and acid‐base balance. For measuring peak βHB levels, participants measured their blood βHB using Abbot OptiumNeo^®^ meters immediately before and 30 min following their third KE drink on their least active day of the week.

For assessing acid‐base balance, venous blood samples were analysed weekly for acid‐base status, electrolytes, glucose, haemoglobin, serum creatinine and urea (Abbot iSTAT^®^ cartridges CG4± and CHEM8±, Abbot Point of Care Inc). Urine was analysed for bilirubin, blood, leukocytes, glucose, ketones, nitrite, pH, protein, specific gravity and urobilinogen (Bayer Multistix^®^ Reagent Strips for Urinalysis, Bayer HealthCare LLC).

Weekly anthropometric measurements (body weight and body composition) and fasting blood and urine samples were collected every 7 days after enrolment. Body composition was measured using four‐point bioelectrical impedance scale (BF508^®^, Omron Electronics Ltd).

Glycaemic control was assessed using (i) blood HbA1c, (ii) blood fructosamine, (iii) mean blood glucose and (iv) blood glucose time in range. Fructosamine was measured using a semi‐automated bench‐top analyser (ABX Pentra^®^, A11A01679). IS‐GM’s were placed on participants 1 week before starting KE supplementation and changed every 2 weeks throughout the 6‐week trial. Target glucose range was 3.9–10 mmol/L (70–180 mg/dl), and time in range was obtained from the Abbot's Libre View^®^ interface.

For assessing cardiovascular risk, HOMA‐IR and 10‐year fatal coronary heart disease risk were calculated using open access calculators available at https://www.dtu.ox.ac.uk/riskengine/download.php. Additionally, plasma total cholesterol, ApoB, triglycerides, HDL, C‐reactive protein, and ALT and AST were measured by the Department of Clinical Biochemistry at the John Radcliffe Hospital, Oxford, UK.

### 2.5 Sample size rationale

Considering the 1‐month duration of the intervention, fructosamine was used for sample size estimation. Other studies report that a 15% decrease in fructosamine is clinically relevant.^17^ Assuming a standard deviation of 20% and given statistical power of 0.80 and alpha of 0.05, 16 participants were required for this study. Sample size calculations were performed using G*Power Software version 3.1.^18^

### 2.6 Analysis

Mean and dispersion values for demographic and biochemical variables were calculated. Two‐tailed Student's *t*‐tests for paired samples were used for comparing means. Significance was taken at *p* < .05. Data are presented as mean ± SD. All calculations were performed using Microsoft Excel^®^. There were fewer than 5% missing data points, which were handled by mean substitution.

## 3 RESULTS

### 3.1 Participant characteristics

Twenty‐three patients were initially enrolled. One participant withdrew after 1 week without reporting any adverse event. Another participant completed 5 weeks of the study but was excluded from the analysis because she was prescribed glucocorticoids during her last week of the intervention. Therefore, twenty‐one participants (14 males and 7 females), aged 45.1 ± 10.8 years, completed the study. Co‐morbidities included hypertension (38%) and dyslipidaemia (28%). Common glycaemic therapies included metformin (90.4%), liraglutide (28%) and empagliflozin (19%) (Table 1).

**TABLE 1:** Participants’ clinical and demographic data

Sex	14 males, 7 females
Age	45 ± 11 years
Hypertension	8 (38%)
Dyslipidaemia	6 (28.5%)
Overweight (BMI>25–30 kg/m^2^)	6 (28.5%)
Obesity (BMI>30 kg/m^2^)	10 (47.6%)
Metformin	19 (90.4%)
Empaglifozin	4 (19%)
Dapaglifozin	3 (14.2%)
Liraglutide	6 (28.5%)
Dulaglutide	2 (9.5%)
Linagliptin	2 (9.5%)
Gliclazide	2 (9.5%)
Acarbose	2 (9.5%)
Pioglitazone	2 (9.5%)
Concomitant medications	1.7 ± 1.0. Median and mode = 1

Note

Data are means ± standard deviation or *n* (%).

### 3.2 Tolerability and adherence

All participants had >90% adherence, defined as consumption of at least 76 of the maximum 84 KE drinks. Thus, the KE drink was well‐tolerated. Adverse symptoms were self‐recorded by participants following each of their drinks and were ranked as ‘mild’, ‘moderate’ or ‘severe’. Overall occurrence of adverse symptoms is expressed relative to the total number, 1,588, of drinks consumed throughout the entire study. Mild nausea (5/1,588 = 0.3%), mild headache (5/1,588 = 0.3%) and mild gastric discomfort (9/1,588 = 0.5%) were the only reported symptoms.

### 3.3 Metabolic outcomes and glycaemic control

Blood electrolytes, acid‐base status and renal function were normal throughout the study. βHB blood concentrations were recorded by each participant 30 min following a 25 ml KE drink and ranged from 3.1 ± 0.5 mM to 3.8 ± 0.7 mM (Table 2).

**TABLE 2:** Weekly blood acid‐base and renal function assessment

	Week 1	Week 2	Week 3	Week 4	Week 5	Week 6
pH	7.39 ± 0.03	7.40 ± 0.03	7.39 ± 0.03	7.40 ± 0.03	7.39 ± 0.04	7.39 ± 0.03
HCO^−^_3_ (mM)	23 ± 2	23 ± 2	22 ± 2	23 ± 2	23 ± 2	23 ± 2
Na^+^ (mM)	139 ± 3	139 ± 3	139 ± 2	139 ± 3	139 ± 4	139 ± 2
K^+^ (mM)	4 ± 0.3	4.0 ± 0.3	4.1 ± 0.3	3.9 ± 0.3	4.0 ± 0.3	3.9 ± 0.3
Cl^−^ (mM)	101 ± 3	100 ± 3	100 ± 3	101 ± 3	100 ± 3	100 ± 3
Anion Gap (mEq/L)	15 ± 4	16 ± 5	17 ± 3	16 ± 5	16 ± 4	16 ± 4
Creatinine (mg/dL)	1.02 ± 0.17	1.00 ± 0.15	1.01 ± 0.13	1.03 ± 0.12	1.02 ± 0.15	1.06 ± 0.17
Lactate (mM)	1.7 ± 0.2	1.6 ± 0.1	1.7 ± 0.1	1.7 ± 0.2	1.6 ± 0.2	1.6 ± 0.1
Systolic pressure (mmHg)	125 ± 12	124 ± 10	124 ± 8	125 ± 8	124 ± 12	126 ± 10
Diastolic pressure (mmHg)	83 ± 10	83 ± 7	83 ± 6	85 ± 8	83 ± 9	84 ± 9
Baseline βHB (mM)	NM	0.1 ± 0.1	0.1 ± 0.1	0.1 ± 0.1	0.1 ± 0.1	NM
30 min βHB (mM)	NM	3.8 ± 0.7	3.4 ± 0.6	3.1 ± 0.5	3.3 ± 0.6	NM

Note

Data are means ± standard deviation for *n* = 21.

Abbreviation: NM, not measured.

All anthropometric and cardiovascular risk markers were not different at baseline and follow‐up (Table 3).

**TABLE 3:** Plasma and anthropomorphic cardiovascular risk markers before and after consuming the ketone monoester

	Baseline	After 28 days
Triglycerides (mM)	1.18 ± 0.57	1.10 ± 0.43
Cholesterol (mM)	5.35 ± 1.3	5.84 ± 1.08
HDL (mM)	1.84 ± 0.54	1.84 ± 0.57
ApoB (mM)	0.9 ± 0.3	0.9 ± 0.3
C‐reactive protein (mg/L)	2.1 ± 0.9	1.9 ± 0.9
Total weight (kg)	96.7 ± 15.7	95.4 ± 15.6
Total body fat %	33.2 ± 6.6	33.0 ± 6.6
Visceral fat %	12.4 ± 2.7	12.3 ± 2.8
ALT (U/L)	41 ± 15	38 ± 17
AST (U/L)	40 ± 14	38 ± 15
NEFA (mM)	1.03 ± 0.19	0.99 ± 0.20
HOMA–IR^a^	1.66 ± 0.37	1.48 ± 0.45
10‐year fatal CHD risk^a^ (%)	7.7 ± 3.3	7.3 ± 3.9

Note

Data are means ± standard deviations. All variables were compared against a baseline using a two‐tailed paired Student's *t*‐test. *p* was >.05 for all comparisons.

^a^ HOMA and 10‐year fatal coronary heart disease risk were calculated using an open access diabetes‐specific calculator available at https://www.dtu.ox.ac.uk/riskengine/download.php

Compared with baseline and follow‐up, the KE intervention caused a significant decrease in both HbA1c, from 61 ± 10 mmol/mol to 55 ± 9 mmol/mol (7.7 ± 0.9% to 7.2 ± 0.9%) (*p* < .01) and fructosamine 335 ± 60 μM to 290 ± 49 μM (*p *< .01). Compared to baseline and follow‐up, exogenous ketosis decreased mean blood glucose from 7.8 ± 1.4 mM to 7.4 ± 1.3 mM (140 ± 23 mg/dL to 133 ± 25 mg/dL) (*p* < .01).

**FIGURE 1:**
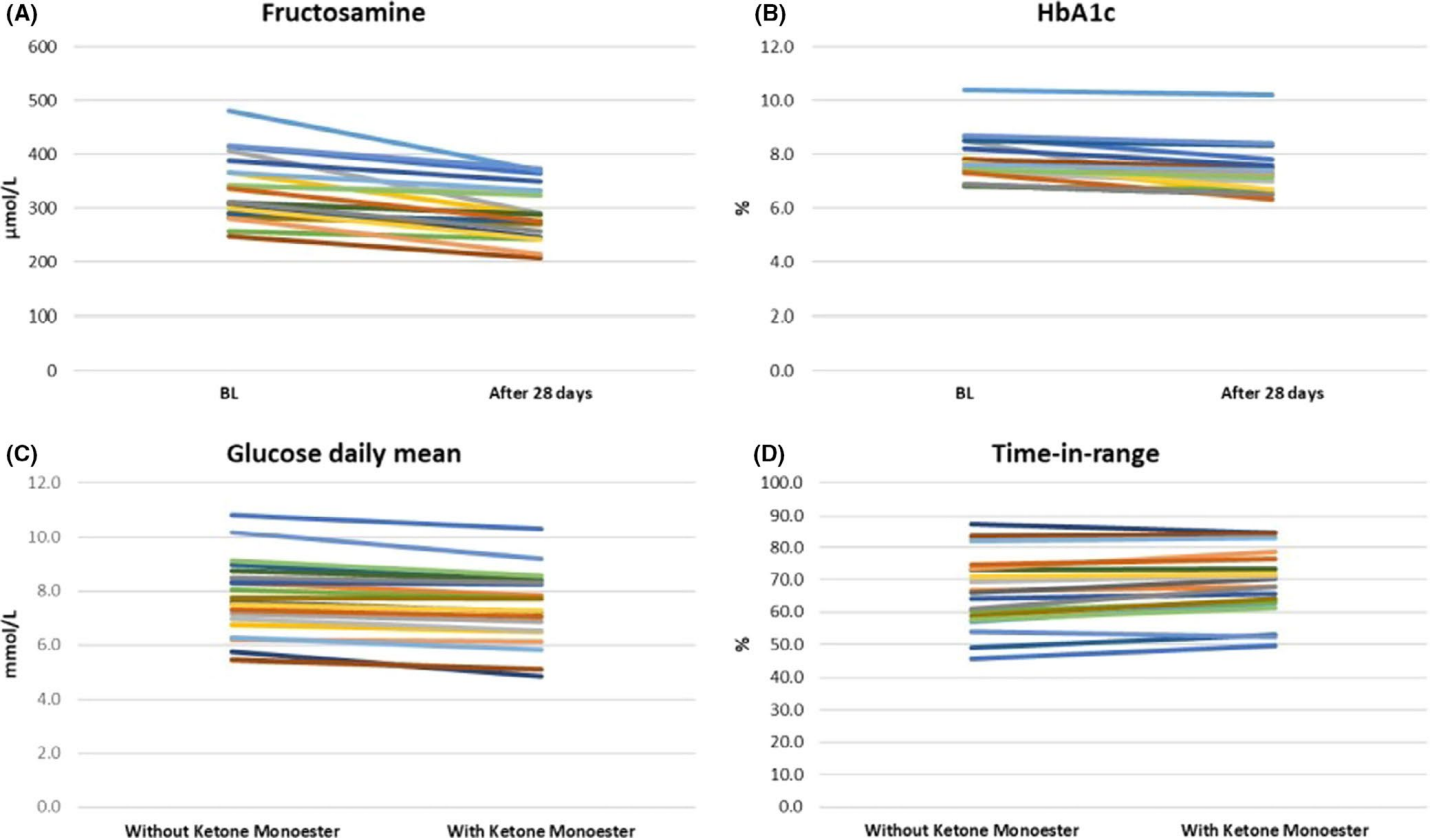
The effect of exogenous ketosis on glycaemic control indicators. (A) Fructosamine change from baseline after 28 days of consuming the ketone monoester. (B) HbA1c change from baseline after 28 days of consuming the ketone monoester. (C) Interstitial glucose daily mean difference between the days without (weeks 1 and 6) and with (weeks 2–5) ketone monoester. (D) Time in range difference between the days without and with ketone monoester. All variables were compared using a two‐tailed paired Student's *t*‐test. *p* was <.01 for all comparisons. *n* = 21

The per cent time in range for the blood glucose readings also improved significantly during the KE intervention from 67 ± 11% to 69 ± 10% (*p* < .01). Time above range decreased from 29 ± 6% to 26 ± 7% (*p* < .01), and time below range did not change significantly 3.5 ± 0.7% to 3.6 ± 0.9% (*p* > .05). Thus, the KE drinks improved all four metrics of glycaemic control in 21 patients with T2D (Figure 1).

## 4 DISCUSSION

The primary purpose of this study was to evaluate the safety and tolerability of KE drinks in patients with T2D. Since no moderate or severe symptoms were recorded, the frequency of any given mild symptoms was 0.5% or less, and as no patient experienced symptomatic hypoglycaemia, it is reasonable to conclude that the KE is safe and well‐tolerated. Furthermore, the extremely low number of even mild symptoms is congruent with participants’ high adherence to the KE drink regimen.

No statistically significant differences were observed in anthropomorphic or cardiovascular risk parameters, other than those related to glycaemic control. This includes no differences in blood pressure, fasting lipid profile, C‐reactive protein and body composition, which contrasts with animal studies that report changes in such metrics.^19^ However, there was a small reduction in total weight of 1.3 kg which could have be greater with a longer‐term intervention, meriting further research. It is worth highlighting that these metrics were followed primary for safety purposes and that this study was not powered, nor intended to look for, changes in these markers. In other words, it is possible that improvements in weight and markers other than those related to glycaemic control may have been observed had the intervention lasted longer than 1 month or had a larger cohort of patients been studied.

Importantly, all four markers of glycaemic control—HbA1c, fructosamine, mean daily blood glucose and time in range—improved during sustained exogenous ketosis. HbA1c is the most widely used and acknowledged glucose control outcome measure in research and clinical practice^20^ so was important to include in this study. However, HbA1c is thought to approximate average blood glucose over 3 months. Therefore, the 6 mmol/mol improvement observed in HbA1c, while significant, underestimates the effect of KE consumption on this parameter. Extrapolating these data to 3 months, the magnitude of the effect of KE would be comparable to the decrease in HbA1c achieved by most currently prescribed oral hypoglycaemic medications for T2D,^21^ but is lower than what has been observed in studies involving ketogenic diets in T2D.^2^

The shorter half‐life of fructosamine, compared to HbA1c, makes it a better metric for glycaemic control in interventions lasting less than 3 months.^22^ The reduction in fructosamine observed in our study, from 335 to 290 μM, is comparable to the effects of moderate to intense exercise interventions. In a study involving eight patients with T2D who exercised at 50%–60% VO_2_ peak for 30–60 min three times per week for 8 weeks, fructosamine decreased by 57 μM^17^ (compared to 45 μM in this study).

As βHB and exercise likely operate to control glycaemia in T2D via complementary mechanisms, the comparison of the KE regimen to the effects of exercise is favourable, not only in terms of the effect of magnitude, but also with respect to the fact that combining KE therapy and exercise may have an additive effect on glycaemic control. Indeed, recent work has shown that KE is also efficacious in improving exercise tolerance in patients with chronic illnesses like Parkinson's disease.^23^

In addition to HbA1c and fructosamine, both blood glucose daily mean values and blood glucose time in range exhibited significant improvements after sustained exogenous ketosis. The finding that all four metrics of glycaemic control reached statistical significance strongly suggests that KE is effectively improves glucose control in patients with T2D.

The main limitations of this study were the lack of a placebo group and the relatively small sample size. A placebo group was not included because the primary purpose of this pilot was to demonstrate the safety and tolerability of KE in patients with T2D. As mentioned before, due to the 1‐month duration of this study, it was not possible to explore potential longer‐term glycaemic benefits from inducing sustained exogenous ketosis, such as a reduction in micro‐ and macrovascular complications. Additionally, we included patients using SGLT2 inhibitors, which have been associated with euglycaemic ketoacidosis. The unlikely, but possible, interaction between these inhibitors and sustained exogenous ketosis cannot be ruled out and merits further research in placebo‐controlled studies with longer follow‐up times and larger sample sizes. Also, we did not closely measure food consumption. The bitter taste of the ketone drink could have reduced appetite and food intake, which would alter glycaemia. However, the bitterness lasts for a few minutes and hunger reduction can also be directly attributed to elevated BHB blood levels.^24^

Adherence was high (>90%) in this study, possibly because all participants had tasted the bitter‐tasting KE drink before being enrolled. However, it is possible that the taste could challenge its routine long‐term consumption in a real‐world setting. Finally, the KE drink is currently expensive on the free market, so it would be important to conduct cost‐benefit studies. Nevertheless, these results are promising and warrant future placebo‐controlled studies with sufficient statistical power to allow treatment‐group comparisons.

## 5 CONCLUSIONS

These results confirm the safety and tolerability of three times daily KE drinks in patients with T2D. The KE drinks improved four markers of glycaemic control: HbA1c, fructosamine, mean glucose and time in range. Randomized clinical trials are needed to evaluate their clinical efficacy.

## CONFLICT OF INTEREST

The authors declare the following financial interests/personal relationships which may be considered as potential competing interests: The intellectual property covering the uses of ketone bodies and ketone esters are owned by Boston Scientific Plc, the University of Oxford and the US National Institutes of Health. Professor Kieran Clarke, as an inventor, will receive a share of the royalties under the terms prescribed by each institution. Professor Kieran Clarke is a director of TdeltaS Ltd, a company spun out of the University of Oxford to develop products based on the science of ketone bodies in human nutrition.

## AUTHOR CONTRIBUTIONS

All authors contributed significantly to the design and execution and reporting of this study. The authors would like to thank Vicky Ball, Poonam Patel, Alison Campbell, Jill Woodford, Julie Jones, Dr Sushma Datla and Dr Petra Hanson, for their invaluable assistance during this study. Dr Adrian Soto‐Mota is the guarantor of this study and takes responsibility for the contents of this article.

## Supporting information

Figure S1

## Data Availability

The data that support the findings of this study are available on request from the corresponding author. The data are not publicly available due to privacy or ethical restrictions.
