# Supplementary figures and images for: Exogenous ketosis in patients with type 2 diabetes: Safety, tolerability and effect on glycaemic control
Source: Endocrinol Diabetes Metab. 2021 May 20;4(3):e00264. doi: 10.1002/edm2.264 (PMC8279633; doi:10.1002/edm2.264)

## Supplemental Figure.

## Study Flowchart

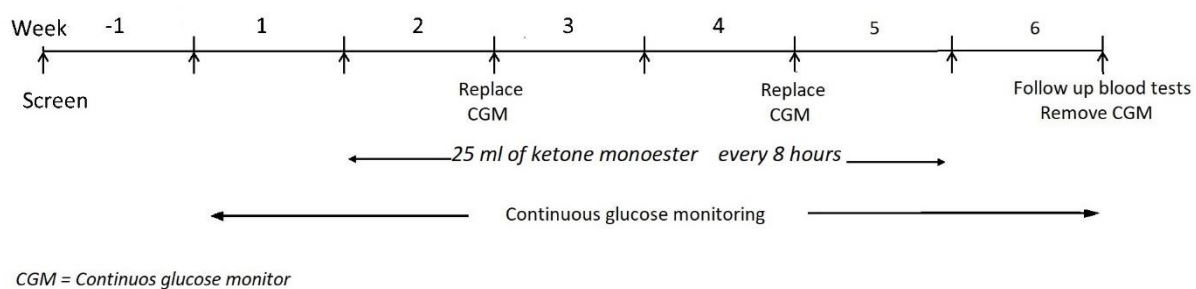

Supplement: Supplementary file 1 — Figure S1 [file EDM2-4-e00264-s001.pdf]
